# Insulin Clearance Is Associated with Hepatic Lipase Activity and Lipid and Adiposity Traits in Mexican Americans

**DOI:** 10.1371/journal.pone.0166263

**Published:** 2016-11-15

**Authors:** Artak Labadzhyan, Jinrui Cui, Miklós Péterfy, Xiuqing Guo, Yii-Der I. Chen, Willa A. Hsueh, Jerome I. Rotter, Mark O. Goodarzi

**Affiliations:** 1 Division of Endocrinology, Diabetes and Metabolism, Department of Medicine, Cedars-Sinai Medical Center, Los Angeles, California, United States of America; 2 Department of Biomedical Sciences, Cedars-Sinai Medical Center, Los Angeles, California, United States of America; 3 Institute for Translational Genomics and Population Sciences, Los Angeles BioMedical Research Institute, Harbor-UCLA Medical Center, Torrance, California, United States of America; 4 Division of Endocrinology, Diabetes and Metabolism, Department of Medicine, The Ohio State University, Columbus, Ohio, United States of America; Medical University of Vienna, AUSTRIA

## Abstract

Reduction in insulin clearance plays an important role in the compensatory response to insulin resistance. Given the importance of this trait to the pathogenesis of diabetes, a deeper understanding of its regulation is warranted. Our goal was to identify metabolic and cardiovascular traits that are independently associated with metabolic clearance rate of insulin (MCRI). We conducted a cross-sectional analysis of metabolic and cardiovascular traits in 765 participants from the Mexican-American Coronary Artery Disease (MACAD) project who had undergone blood sampling, oral glucose tolerance test, euglycemic-hyperinsulinemic clamp, dual-energy X-ray absorptiometry, and carotid ultrasound. We assessed correlations of MCRI with traits from seven domains, including anthropometry, biomarkers, cardiovascular, glucose homeostasis, lipase activity, lipid profile, and liver function tests. We found inverse independent correlations between MCRI and hepatic lipase (P = 0.0004), insulin secretion (P = 0.0002), alanine aminotransferase (P = 0.0045), total fat mass (P = 0.014), and diabetes (P = 0.03). MCRI and apolipoprotein A-I exhibited a positive independent correlation (P = 0.035). These results generate a hypothesis that lipid and adiposity associated traits related to liver function may play a role in insulin clearance.

## Introduction

The pathogenesis of type 2 diabetes involves an interplay of genetics and environment that results in insulin resistance and beta cell dysfunction. Insulin resistance is associated with obesity, elevated free fatty acids, and genetic defects [1]. Maintenance of normal glucose tolerance in the face of insulin resistance is dependent on the compensatory increase in circulating insulin levels to overcome insulin resistance; when this compensation is insufficient, diabetes develops. Compensatory hyperinsulinemia is not only related to increased insulin secretion by pancreatic β-cells but also to reduction in insulin clearance [2].

Insulin clearance is mainly carried out by the liver and kidneys as well as by uptake and degradation peripherally by insulin sensitive tissues [3]. Factors influencing insulin clearance include obesity, distribution of adiposity, fatty acids, glucose, catecholamines, and other hormones including growth hormone and sex hormones [3–5].

The metabolic clearance rate of insulin (MCRI) has been shown to be a highly heritable trait that can influence the risk of developing diabetes [6]. A genome-wide association study (GWAS) found that variants near the *IDE* and *HHEX* genes were associated with risk of type 2 diabetes [7]. While follow-up studies suggested variation in this locus may affect insulin secretion [8, 9], an effect on insulin clearance remains possible. A Metabochip study found that seven loci previously associated with type 2 diabetes were associated with insulin clearance [10]. To date, the only published GWAS of insulin clearance identified a variant in the *GNA15* gene that nearly met genome-wide significance [11].

In a study of Hispanics and African Americans, lower insulin clearance at baseline was associated with a higher risk of incident diabetes at five years of follow-up [12]. However, an independent association has been difficult to establish given the many possible confounding factors and the lack of multivariate studies looking at traits across a wide spectrum of categories.

Our aim was to determine the association with MCRI of metabolic and cardiovascular traits within several phenotypic domains and to identify which of these traits are independently correlated with insulin clearance. By identifying unique traits associated with MCRI, our hope was also to gain insight on the underpinnings of insulin clearance that to date have not been clearly elucidated. The breadth of traits examined allowed the generation of new hypotheses.

## Materials and Methods

### Subjects

Metabolic and cardiovascular phenotypes were measured in subjects participating in the UCLA/Cedars-Sinai Mexican-American Coronary Artery Disease (MACAD) project, a study of Mexican-American families living in Los Angeles [6]. Subjects had to report at least three grandparents of Mexican origin to be classified as Mexican and qualify for the study. In the current report, we studied 765 subjects from 193 families (57% female and 43% male) with insulin clearance values, consisting of adult offspring (age 18 or older) of probands with coronary artery disease, and the spouses of those offspring (if available). By design, detailed phenotyping was performed only in the offspring and their spouses, who were free of overt cardiovascular and metabolic disease, to avoid secondary changes in phenotype caused by overt disease. Patients were free of major medical illness and none were taking glucocorticoids or antihyperglycemic agents that could affect glucose homeostasis.

### Phenotyping procedures

Subjects underwent a three-day phenotyping protocol that included anthropometry, biomarkers, cardiovascular traits, glucose homeostasis indices, lipase activity, lipid parameters, and liver function tests. On one day, subjects gave fasting blood and underwent a 75 g oral glucose tolerance test (OGTT). On a different day, common carotid artery intima-media thickness (IMT) was measured by B-mode ultrasound and body fat distribution was assessed by dual-energy X-ray absorptiometry (DXA) scan. On a further day, subjects underwent a euglycemic-hyperinsulinemic clamp.

Fasting lipid parameters examined included total cholesterol, low-density lipoprotein cholesterol (LDL-C), high-density lipoprotein cholesterol (HDL- C), triglycerides (TG), free fatty acids (FFA), and apolipoproteins (apoA-I, apoA-II, apoB). Fasting biomarkers included C-reactive protein (CRP), adiponectin, and plasminogen activator inhibitor-1 (PAI-1) levels. Liver function tests included aspartate aminotransferase (AST) and alanine aminotransferase (ALT).

Homeostasis model assessment of steady state beta cell function (HOMA-%B) was derived from fasting glucose and insulin using the updated HOMA2 calculator at https://www.dtu.ox.ac.uk/homacalculator/. Insulinogenic index at 30 minutes (IGI30, pmol·l^-1^ · mmol^-1^·l), a commonly used index of acute insulin secretion, was calculated from the OGTT data as the change in insulin (0 to 30 min) divided by change in glucose (0 to 30 min). At the beginning of the clamp, a priming dose of human insulin (Novolin, Clayton, NC) was given. This was followed by infusion of insulin for 120 minutes at a constant rate (60 mU·m^-2^·min^-1^) with the goal of achieving steady state plasma insulin levels of 100 mIU/ml or greater [13, 14]. Guided by blood sampling every 5 minutes, the rate of a simultaneous 20% dextrose infusion was adjusted to maintain plasma glucose concentrations at 95 to 100 mg/dl. The glucose infusion rate (M value, μmol·m^-2^·min^-1^) during the last 30 minutes of steady-state glucose and insulin levels reflects glucose uptake by all tissues of the body (mainly insulin-mediated glucose uptake in muscle) and is directly correlated with tissue insulin sensitivity [14]. Often, an insulin sensitivity index (M/I, mg·m^-2^·min^-1^·μIU^-1^·mL) is calculated as M divided by the steady state plasma insulin level (I). In this study, to clearly distinguish between insulin sensitivity and insulin clearance in multivariate analyses, we relied on M as an approximation for insulin sensitivity in our primary analyses because the calculations of M/I and insulin clearance both use steady-state insulin in the denominator. The metabolic clearance rate of insulin (MCRI, mL·m^-2^·min^-1^) was calculated as the insulin infusion rate divided by the insulin concentration during the steady state of the euglycemic clamp, as previously described [6, 14].

Cardiovascular phenotypes consisted of systolic and diastolic blood pressure (SBP and DBP), pulse, and carotid intima-media thickness (IMT) [15]. At the University of Southern California Atherosclerosis Research Unit, carotid artery images were acquired by high-resolution B-mode ultrasound using the Toshiba SSH-140A ultrasound system with a 7.5-MHz probe [16]. The IMT measure corresponds to the distance between the blood-intima and media-adventitia echoes obtained at the right distal common carotid artery [16]. The IMT values are reported as the average of 80 to 100 individual measurements made along 1 cm of the right distal common carotid artery [16].

Lipase activity included hepatic lipase (HL) and lipoprotein lipase measurements after heparin administration, as previously described [17]. Also tested were total fat mass and total lean mass measured by DXA, as well as anthropometric indices (BMI, height, weight, waist circumference, hip circumference) [18].

### Ethics statement

All studies were approved by Human Subjects Protection Institutional Review Boards at UCLA and Cedars-Sinai Medical Center. All subjects gave written informed consent prior to participation.

### Statistical analysis

When necessary, log‐transformed (hip circumference, BMI, adiponectin, CRP, IMT, pulse, apoA-I, apoA-II, apoB, HDL-C, TG, ALT, AST, HOMA-%B, IGI30, 2-hr plasma glucose during OGTT, 2-hr plasma insulin during OGTT) or square‐root‐ transformed (total fat mass, PAI-1, FFA, M value, hepatic lipase, lipoprotein lipase, MCRI) trait values were used to normalize the distribution for statistical analyses. Categorical traits (sex and diabetes status) were coded as 0 and 1 prior to inclusion in analyses. Traits were categorized into seven domains: anthropometry, biomarkers, cardiovascular, glucose homeostasis, lipase activity, lipid profile, and liver function tests.

Generalized estimating equations (GEE) were used to assess the effects of single traits (univariate analyses) or joint effects of multiple traits (multivariate analyses) on MCRI, adjusting for familial relationships. The weighted GEE1 [19] was computed assuming an exchangeable correlation structure and using the sandwich estimator of the variance to account for familial correlation present in family data. GEE was used to derive standardized regression coefficients, which in any one regression equation are measured on the same scale, with a mean of zero and a standard deviation of one. They are then directly comparable to one another, with the largest coefficient indicating which independent variable has the greatest association with the dependent variable.

**Fig 1:**
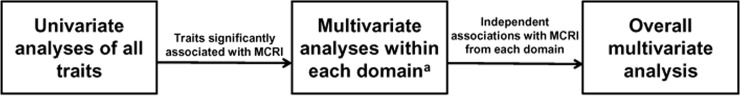
Three stages of statistical analyses. All analyses were done with MCRI as the dependent variable. Traits that were significant in univariate analysis were included in the multivariate analyses within each domain. The overall multivariate analysis included traits that were significant from each of the within-domain analyses. ^a^ Domains include anthropometry, biomarkers, cardiovascular, glucose homeostasis, lipase activity, lipid profile, and liver function tests.

Analyses were carried out in three stages. First, univariate analyses of all traits of interest versus MCRI was carried out. Next, within the trait domains with more than one variable significantly associated with MCRI, multivariate analysis was conducted with MCRI as the dependent variable. These multivariate analyses were carried out separately for each trait domain, using only traits that were significant in the univariate analysis as independent variables. Finally, an overall multivariate analysis analyzed the associations with MCRI of the significant traits from each of the within-domain analyses. The three stages of the analysis scheme are shown in Fig 1. Given that 34 variables were tested in the first stage, only variables meeting a multiple testing corrected P value cutoff of 0.0015 (0.05/34) were advanced to downstream analyses. In the multivariate analyses, P values of <0.05 were considered significant.

To assess for multicollinearity within the regression models, variance inflation factors (VIF) were calculated. VIF less than 5 are acceptable while VIF above 10 indicate poorly estimated regression coefficients due to multicollinearity.

Finally, median MCRI values by quartile of the traits significantly associated with MCRI in the overall multivariate analysis are presented to convey the effect sizes and pattern of the associations. Untransformed MCRI values are presented for ease of clinical interpretation.

## Results

The clinical characteristics of the 765 subjects are shown in Table 1. Participants had a median age of 34 years and 57% were women. Subjects were generally healthy with normal range median systolic and diastolic blood pressure, pulse, lipid profile, and liver function tests. Only 3% of participants had diabetes as diagnosed by oral glucose tolerance testing.

**Table 1:** Clinical characteristics and univariate analyses of all traits.

Domain/Trait	Median (IQR)^a^	r	P-value
MCRI (mL·m^-2^·min^-1^)	468.8 (143.6)	n/a	n/a
**ANTHROPOMETRY**			
Age (years)	34.0 (13.0)	0.0095	0.83
Sex (% female)	56.3	-0.050	0.45
Waist circumference (cm)	91.8 (16.0)	-0.26	<0.0001
Hip circumference (cm)	104.0 (12.5)	-0.24	<0.0001
Body mass index (BMI) (kg/m^2^)	28.2 (6.3)	-0.27	<0.0001
Height (cm)	162.0 (13.0)	-0.029	0.41
Total fat mass (kg)	23.9 (11.1)	-0.29	<0.0001
Total lean mass (kg)	50.6 (18.8)	-0.12	0.0014
**BIOMARKERS**			
Plasminogen activator inhibitor-1 (PAI-1) (pmol/l)	613.5 (536.5)	-0.15	<0.0001
Adiponectin (μg/ml)	6.9 (4.3)	0.080	0.044
C reactive protein (CRP) (nmol/l)	13.3 (19.0)	-0.057	0.24
**CARDIOVASCULAR**			
Carotid intima media thickness (mm)	0.6 (0.1)	0.0003	0.99
Systolic blood pressure (SBP) (mmHg)	112.3 (18.3)	-0.0086	0.79
Diastolic blood pressure (DBP) (mmHg)	66.7 (12.7)	-0.014	0.71
Pulse (beats/min)	69.3 (14.3)	-0.042	0.20
**GLUCOSE HOMEOSTASIS**			
M value (insulin sensitivity) [μmol·min^-1^·m^-2^]	1267.8 (830.4)	0.14	0.0003
HOMA-%B (%)	120.0 (50.1)	-0.33	<0.0001
Insulinogenic index at 30 minutes (pmol·l^-1^ · mmol^-1^·l)	140.5 (140.5)	-0.19	0.0002
Fasting glucose (mmol/l)	5.14 (0.72)	0.0088	0.83
2-hour glucose (mmol/l)	6.16 (2.39)	-0.042	0.20
2-hour insulin (pmol/l)	345.0 (405.0)	-0.20	<0.0001
Diabetes (%)	2.6	-0.33	0.0059
**LIPASE ACTIVITY**			
Hepatic lipase (nmol·ml^-1^·min^-1^)	27.8 (19.2)	-0.17	<0.0001
Lipoprotein lipase (nmol·ml^-1^·min^-1^)	28.5 (32.2)	-0.033	0.47
**LIPID PROFILE**			
Total cholesterol (mg/dl)	4.58 (1.19)	-0.036	0.37
Low density lipoprotein cholesterol (LDL-C) (mmol/l)	2.75 (0.97)	-0.054	0.18
High density lipoprotein cholesterol (HDL-C) (mmol/l)	1.16 (0.39)	0.14	0.001
Triglycerides (mmol/l)	1.23 (1.03)	-0.10	0.0065
Apolipoprotein A type I (apoA-I) (g/dl)	1.11 (0.23)	0.15	0.0002
Apolipoprotein A type II (apoA-II) (g/dl)	0.31 (0.06)	0.017	0.69
Apolipoprotein B (apoB) (g/dl)	0.85 (0.32)	-0.081	0.035
Non-esterified fatty acids (mmol/l)	0.65 (0.31)	-0.011	0.74
**LIVER FUNCTION TESTS**			
Alanine transaminase (ALT) (μkat/l)	0.38 (0.28)	-0.17	<0.0001
Aspartate transaminase (AST) (μkat/l)	0.38 (0.15)	-0.13	0.001

^a^Quantitative traits are presented as median (interquartile range (IQR)). Categorical traits (sex, diabetes) are presented as percent.

Traits in the anthropometry domain that were significantly correlated with MCRI in the univariate analysis included waist, hip, BMI, total fat mass, and total lean mass (Table 1). In multivariate analysis, only fat mass remained significant (Table 2). Traits with significant correlation within the glucose homeostasis group were M value, IGI30, insulin at 120 minutes of the OGTT, HOMA-%B, and diabetes status, the latter two of which also remained significant in multivariate analysis in this domain. Only hepatic lipase was significantly correlated with MCRI from the lipase activity domain, with lipoprotein lipase not showing significant correlation. HDL-C, TG, and apoA-I were all significant in univariate analysis, but only apoA-I remained significant in multivariate analysis. Only biomarker PAI-1 was significant in univariate analysis. Both ALT and AST were significant in univariate analysis but only ALT remained significant in multivariate analysis. IMT, SBP, DBP, and pulse were not significant in univariate analysis.

**Table 2:** Multivariate analyses within each domain.

Domain/Trait	Standardized coefficient	Standard error	95% confidence interval		P-value
**ANTHROPOMETRY**					
BMI	0.019	0.072	-0.12	0.16	0.79
Waist	-0.094	0.074	-0.24	0.051	0.20
Hip	0.073	0.066	-0.056	0.201	0.27
Total fat mass	-0.230	0.079	-0.45	-0.14	0.0002
Total lean mass	-0.071	0.053	-0.18	0.034	0.183
**GLUCOSE HOMEOSTASIS**					
M value	-0.0023	0.054	-0.11	0.10	0.97
IGI30	-0.079	0.046	-0.17	0.012	0.09
Diabetes	-0.38	0.13	-0.63	-0.14	0.0024
HOMA-%B	-0.29	0.050	-0.39	-0.19	<0.0001
2-hour insulin	-0.058	0.054	-0.16	0.048	0.29
**LIPID PROFILE**					
HDL-C	-0.024	0.067	-0.16	0.11	0.72
Triglycerides	-0.11	0.045	-0.19	-0.017	0.019
ApoA-I	0.16	0.060	0.041	0.28	0.008
**LIVER FUNCTION TESTS**					
ALT	-0.19	0.059	-0.30	-0.071	0.0015
AST	0.024	0.063	-0.10	0.15	0.71

Variance inflation factors ranged from 2.44 to 5.16 for anthropometry; 1.07 to 2.08 for glucose homeostasis; 1.49 to 3.52 for lipid profile; and 2.67 to 2.89 for liver function tests.

In overall multivariate analysis, only diabetes, total fat mass, hepatic lipase, ALT, HOMA-%B, and apoA-I were significant (Table 3). Comparison of standardized coefficients allowed determination of the relative strength of each trait’s association with MCRI (listed strongest to weakest): diabetes (-0.28, P = 0.030), HOMA-%B (-0.22, P = 0.0002), hepatic lipase (-0.17, P = 0.0004), total fat mass (-0.12, P = 0.014), ALT (-0.12, P = 0.0045), and apoA-I (0.11, P = 0.035).

**Table 3:** Overall multivariate analysis.

Trait	Standardized coefficient	Standard error	95% confidence interval		P-value
Hepatic lipase	-0.17	0.048	-0.26	-0.077	0.0004
HOMA-%B	-0.22	0.059	-0.33	-0.10	0.0002
ALT	-0.12	0.040	-0.19	-0.036	0.0045
Total fat mass	-0.12	0.050	-0.22	-0.025	0.014
Diabetes	-0.28	0.13	-0.54	-0.027	0.030
ApoA-I	0.11	0.050	0.0075	0.21	0.035
PAI-1	0.0017	0.040	-0.076	0.080	0.97

Variance inflation factors ranged from 1.02 to 2.24.

To provide information on the clinical relevance of the final associations, Table 4 displays median MCRI values by quartile of the five quantitative traits that were significant in the overall multivariate analysis. Median MCRI was 444.9 mL·m^-2^·min^-1^ (interquartile range 102.1) in the subjects with diabetes and 470.0 mL·m^-2^·min^-1^ (IQR 145.2) in the subjects without diabetes.

**Table 4:** Median MCRI values by quartile of significant traits from the overall multivariate analysis.

	Quartile 1	Quartile 2	Quartile 3	Quartile 4
Hepatic lipase	489.1 (146.0)	474.9 (132.1)	473.7 (135.2)	418.1 (116.1)
HOMA-%B	508.5 (130.3)	503.6 (158.1)	471.2 (131.0)	414.8 (138.3)
ALT	492.6 (146.7)	476.2 (133.0)	466.3 (152.7)	438.5 (150.6)
Total fat mass	506.5 (145.9)	477.5 (147.3)	462.7 (131.7)	443.4 (153.1)
ApoA-I	454.5 (116.6)	466.3 (141.2)	476.4 (125.8)	508.5 (144.5)

MCRI values (mL·m^-2^·min^-1^) are presented as median (interquartile range).

## Discussion

In the current study of a wide spectrum of traits from Mexican-American families, we identified diabetes, insulin secretion, hepatic lipase, total fat mass, ALT, and apolipoprotein A-I as independent factors associated with insulin clearance.

To our knowledge, our study is the first to examine the direct association between hepatic lipase activity and insulin clearance. HL plays a key role in HDL metabolism by hydrolyzing triglycerides and phospholipids and helping produce smaller, more dense HDL particles [20]. It also helps facilitate binding and uptake by liver of cholesteryl esters from lipoproteins [21].

Increased HL activity has been associated with increased intra-abdominal fat content, obesity, insulin resistance, and diabetes [22, 23]. HL activity has also been shown to be positively correlated with severity of hepatic steatosis in non-alcoholic fatty liver disease [24]. Several studies have shown an inverse relationship between insulin clearance and liver fat content [25, 26]. In a large cohort of patients with nonalcoholic fatty liver disease and biopsy proven nonalcoholic steatohepatitis, mild increase in liver fat was associated with significantly impaired hepatic and whole body-insulin clearance [25].

Whether the hyperinsulinemic state seen in fatty liver is related to HL has not been studied. It may be that hepatic lipase’s influence on insulin clearance is related to its influence on the fat content of the liver. Given that increased ALT is a frequent marker of fatty liver disease, our finding of significant association between ALT and insulin clearance also supports this hypothesis. If insulin promotes hepatic lipase activity (as suggested by some studies but not others [27]), the negative relationship between insulin clearance and hepatic lipase activity may be related to higher insulin levels in individuals with lower insulin clearance. It is also possible that lower insulin clearance and increased hepatic lipase activity are consequences of fatty liver.

Liver function enzymes such as ALT and γ‐glutamyltransferase (GGT) have been shown to predict incident type 2 diabetes [28]. Though the exact pathophysiology of this association is not fully understood, it is likely related to the liver’s significant role in glucose production and insulin metabolism [3]. Similar to our observations, other studies in healthy nondiabetic populations have demonstrated inverse relationships between ALT and endogenous clearance of insulin and/or hepatic insulin extraction [12, 29]. The relationship between impaired liver function and insulin clearance does not seem to be related to decreased insulin‐degrading activity [30, 31] and warrants further investigation.

We observed a positive correlation between apolipoprotein A-I and MCRI. This correlation has not been previously explored and may provide a possible contributing mechanism in the pathogenesis of diabetes. ApoA-I is the major protein component of HDL and helps facilitate the process of reverse cholesterol transport [32, 33]. One explanation for the positive correlation in our study may be related to the effect of apoA-I on insulin secretion rather than a direct effect on insulin clearance [34]. However, both apoA-I and HOMA-%B remained significant in our overall multivariate analysis, which suggests that the effect of apoA-I on insulin clearance is independent of insulin secretion (with the caveat that HOMA-%B may not perfectly represent insulin secretion). Given the opposite directions of association seen between insulin resistance and insulin secretion and between insulin resistance and insulin clearance [2], the inverse relationship of HOMA-%B and MCRI in our study was expected.

Similar to our hypothesis for HL, the association of apoA-I with MCRI may be mediated through effects on liver fat content. ApoA-I deficient mice exhibited increased diet-induced hepatic triglyceride deposition, with gene transfer with apoA-I(Milano) resulting in significant reduction in hepatic triglyceride content and improvement in hepatic histology [35]. A similar inverse relationship of apoA-I and fatty liver was shown in patients with nonalcoholic steatohepatitis [36].

Though HDL-C and triglycerides were significantly associated with MCRI in univariate analysis, the significance was lost in multivariate analysis. Other studies have shown a positive correlation between HDL-C and MCRI; however, the multivariate models in these studies did not include apoA-I [12, 37]. The physical relationship of apoA-I and HDL may explain why HDL-C level did not correlate with MCRI when analyzed jointly.

The presence of diabetes correlated negatively with insulin clearance in the overall multivariate model. This is consistent with prior studies linking decreased insulin clearance with an adverse metabolic profile and increased risk of diabetes [4, 12, 37–39].

In the anthropometric domain of traits, we found an inverse correlation between total fat mass and MCRI. Previous studies have shown a significant negative correlation between BMI and insulin clearance [39, 40]; however, a recent study found no significant association between BMI and insulin clearance after accounting for insulin resistance [38]. A limitation in these studies has been the paucity of anthropometric traits evaluated. The findings from our multivariate analysis suggest that total fat mass is a better predictor of insulin clearance than BMI, waist circumference, or total lean mass.

Our study advances the field of the physiology of insulin clearance. While large scale studies have investigated the relationship of anthropometric, cardiovascular, glucose homeostasis, and lipid trait domains with insulin clearance derived from the frequently sampled intravenous glucose tolerance test [12, 37, 39], until now no extensive study looking at multiple domains had been performed using euglycemic clamp-derived insulin clearance. The availability of multiple traits in the MACAD cohort allowed interrogation of several key domains, including commonly available traits (e.g., BMI) as well as more unique traits (e.g., total fat mass). To our knowledge, this is the first euglycemic clamp study to examine the relationship of apolipoproteins, C-reactive protein, plasminogen activator inhibitor-1, and hepatic and lipoprotein lipase activities with insulin clearance. Most euglycemic clamp publications concerning insulin clearance have been conducted in small sample sizes, often less than 50 and rarely greater than 100 subjects. Our cohort of over 750 healthy subjects who underwent detailed physiologic phenotyping therefore represents a valuable contribution to this field. Our novel results include the identification within each trait domain of the traits independently associated with insulin clearance (e.g., total fat mass but not total lean mass). We also uniquely describe the independent negative associations of hepatic lipase activity and apolipoprotein A-1 with insulin clearance.

Though the findings in our study are compelling, the results should be interpreted carefully. Limitations include the cross-sectional design and lack of longitudinal follow-up data, which limits our ability to infer causality. Because our measurement of MCRI reflects whole body insulin clearance, we were not able to separately study components of insulin clearance such as first pass hepatic extraction, renal clearance or peripheral tissue clearance. Liver fat was not measured in MACAD and may be an important consideration for future studies. Given that ALT is a poor surrogate for liver disease (e.g., patients with advanced non-alcoholic liver disease may have normal circulating liver enzymes), imaging studies to quantify liver fat would have allowed us to more accurately evaluate the role of hepatic steatosis in insulin clearance. Given that the kidneys also clear insulin, lack of renal functions tests is a limitation that should be addressed in future studies. As the current results were generated in a cohort of Mexican Americans, whether the findings apply to other ethnic groups is unknown.

Insulin clearance is an important component of insulin metabolism, with a decrease in clearance levels predicting increased incidence of type 2 diabetes [12]. We demonstrated for the first time that hepatic lipase was significantly and inversely associated with insulin clearance rate. The association between apoA-I and insulin clearance was also a novel finding. Further studies are needed to clarify the mechanisms of the above markers in insulin clearance and can provide potential therapeutic targets for prevention and treatment of diabetes. Our hypothesis-generating findings suggest mechanisms whereby lipid and adiposity factors related to liver function and structure may play important roles in insulin clearance.
